# A novel frameshift variant in the *SLC2A1* gene causing a mild phenotype of GLUT1 deficiency syndrome: case report

**DOI:** 10.1016/j.ymgmr.2024.101164

**Published:** 2024-11-19

**Authors:** Lívia Maria Ferreira Sobrinho, Thiago Oliveira Silva, Lilia Farret Refosco, Soraia Poloni, Fabiano Oliveira Poswar, Carolina Fischinger Moura de Souza, Fernanda Sperb-Ludwig, Ida Vanessa Doederlein Schwartz

**Affiliations:** a Postgraduate Program in Genetics and Molecular Biology, Universidade Federal do Rio Grande do Sul (UFRGS), Porto Alegre, RS, Brazil; b Department of Pediatrics, School of Medicine, Federal University of Minas Gerais, Minas Gerais, Brazil; c Medical Genetics Service, Hospital de Clínicas de Porto Alegre, Rio Grande do Sul, Brazil; d Clinical Research Center, Hospital de Clínicas de Porto Alegre, Rio Grande do Sul, Brazil; e BRAIN Laboratory, Experimental Research Center, Hospital de Clínicas de Porto Alegre, Rio Grande do Sul, Brazil; f InRaras – Instituto Nacional de Ciencia e Tecnologia em Doenças Raras, Porto Alegre, Rio Grande do Sul, Brazil

**Keywords:** Molecular diagnosis, GLUT1 deficiency syndrome, *SLC2A* gene, Neuropsychomotor developmental delay, Ketogenic diet

## Abstract

Glucose transporter type 1 deficiency syndrome (GLUT1) is a genetic condition, most often of autosomal dominant inheritance, and corresponds to a broad spectrum of signs and symptoms due to hypoglycorrhachia, which include seizures, delay in neuropsychomotor development, intellectual disability, movement disorders, dysarthria and postnatal microcephaly. The severity of symptoms are variable. Symptomatic treatment consists of the ketogenic diet, which allows energy supply to the brain through sustained and continuous ketosis. In this study, we report a novel heterozygous frameshift variant (c.855_856insTT; p.Gly286Leufs*55) in the *SLC2A1* gene in a preschool Brazilian child with atypical phenotype of GLUT1 deficiency syndrome, characterized by ataxia and mild speech delay. Our study enriches the *SLC2A1* gene mutation spectrum and emphasizes the importance of molecular genetic studies for screening patients with neuropsychomotor developmental delay.

**Sentence take-home message (synopsis) of the article**: The study enriches the *SLC2A1* gene mutation spectrum and emphasizes the importance of molecular genetic studies for screening patients with neuropsychomotor developmental delay.

## 1 Introdution

GLUT1 Deficiency Syndrome is caused by pathogenic variants in the *SLC2A1* gene. The *SLC2A1* gene (OMIM * 138140) is located on chromosome 1p34.2 and has ten coding exons for the main glucose transporter in the brain, placenta and erythrocytes, GLUT1 [1]. The most common form of inheritance of this syndrome is autosomal dominant, with 90 % resulting from *de novo* variants. Rarely, GLUT1 Deficiency Syndrome can be inherited in an autosomal recessive manner [2].

Glucose metabolism is fundamental for neural activity. Normally, glucose crosses the blood-brain barrier through facilitated diffusion using GLUT1 transporters. The normal glucose level in the CSF should be higher than 2/3 of the blood sugar level. Pathogenic variants in the *SLC2A1* gene lead to impaired expression and loss of function of these brain receptors, and consequently impaired glucose and brain energy [1].

Most clinical manifestations are due to brain glucose deficiency, leading to a wide spectrum of clinical manifestations [3]. About 90 % of those affected will present the classic symptoms of the disease, characterized by seizures starting before 2 years old, delay in neuropsychomotor development, hypotonia, dysarthria, postnatal microcephaly and movement disorders such as dystonia, ataxia and chorea. However, 10 % of patients have atypical symptoms, characterized by mild delay in neuropsychomotor development, intermittent ataxia, choreoathetosis, dystonia and hemiplegia [3].The ketogenic diet is highly effective in controlling seizures, movement disorders and acquiring neuropsychological skills and is generally well tolerated by patients [4,5].

The objective of this study is to report the clinical and genotypic findings of a preschool child Brazilian patient with a novel variant in the *SLC2A1* gene and an atypical phenotype of GLUT1 deficiency syndrome.

## 2 Case report

A male Brazilian patient, 4.2 years old, single child of a non-consanguineous and healthy couple. The mother is 37 years old and the father is 38 years old. There were no significant clinical complications during pregnancy. He was born by vaginal delivery at the gestational age of 39 weeks. Birth weight was 3530 g (64 nd percentile), length 49 cm (29nd percentile) and head circumference 34 cm (36nd percentile). Apgar 10/10. There were no neonatal complications.

The patient presented head support at three months, sat without support at 7 months and walked at 1.3 years old, when he began to show signs of ataxic gait and for this reason he began follow-up with pediatric neurology. At 2 years of age, he was still unable to say a single word and was therefore suspected of having autism spectrum disorder. The patient did not present stereotypies or characteristic behavioral changes. He was evaluated by neuropsychologists who ruled out the diagnosis.

The electroencephalogram was normal and magnetic resonance imaging of the brain revealed small non-confluent hyperintense images on T2/Flair in the deep periventricular and subcortical white matter of the cerebral hemispheres.

**Fig. 1:**
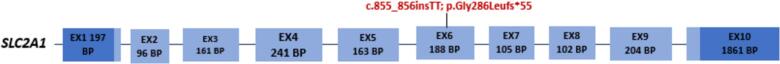
Schematic representation location of the c.855_856insTT frameshift variant; p.Gly286LeufsTer55 in the *SLC2A1* gene (ENST00000426263).

At the age of 3, he was evaluated by a geneticist who requested complete exome sequencing, which showed a heterozygous variant in the *SLC2A1* gene (ENST00000426263):c.855_856insTT; p.Gly286LeufsTer55 (Fig. 1)*.* The segregation of the variant in the parents showed that it was a *de novo variant*. This variant is located in exon 6 and promotes the replacement of the amino acid glycine in codon 286 by leucine and a change in the reading frame from this point on, with the consequent creation of a premature stop codon of protein translation (p.Gly286Leufs*55). This is a null variant in a gene where loss of function is a known mechanism of disease. This variant has not been previously reported in gnomAD or has not previously been associated with a disease phenotype based on ClinVar or published literature. *In silico* prediction of Nonsense mRNA Decay (NMD) suggests that this variant would cause its major affected transcript (ENST00000426263) to be targeted for degradation. According to criteria of the American College of Medical Genetics and subsequent refinements proposed by the Clinical Genome Resource, this variant can be classified as pathogenic (PVS1_very strong and PM2_support) [6].

To assess whether the patient's phenotype was highly specific for GLUT1 Deficiency Syndrome, CSF glucose analysis was performed and showed hypoglycorrhachia [CSF glucose ratio (32 mg/dL) / plasma glucose (75 mg/dL):0.42]. Cell count and protein were normal in CSF. Thus, it is concluded that the variant found in the *SLC2A1* gene is associated with a clinical and biochemical phenotype highly suggestive of GLUT1 deficiency syndrome.

At 3.1 years old a ketogenic diet was started on an outpatient basis. The patient responded well to the diet and there were no reports of adverse effects. He is currently on a 13-month classic ketogenic diet at a 2.5:1 ratio. He is keeping ketones between 3.5 and 5.5 mmol/L. He presents anthropometric data appropriate for his age: weight 19 kg (92nd percentile), height 107 cm (92nd percentile), head circumference (78th percentile). The patient is forming complete sentences, has mild dysarthria, and has demonstrated significant improvement in ataxia as assessed by comparative physical examinations at 3-month intervals. He is attending a regular school and has no learning deficit.

## 3 Discussion

The diagnosis of GLUT1 deficiency syndrome can be confirmed in individuals who present the following findings: suggestive clinical features, hypoglycorrhachia with normal plasma glucose (CSF/plasma glucose ratio less than 0.45) and the identification of a pathogenic variant in heterozygosis (or rarely, homozygosis) in the *SLC2A1* gene [7]*.* Here, we describe a patient who fulfills the three diagnostic criteria of GLUT1 deficiency syndrome. The patient had a novel heterozygous frameshift variant in the *SLC2A1* gene, c.855_856insTT (p.Gly286LeufsTer55.

An association between the specific type of pathogenic variant in *SLC2A1* gene and phenotypic expressivity has been previously reported in the literature. Missense variants were predominantly associated with a mild phenotypic spectrum, while nonsense, frameshift and exon deletion variants were associated with more severe clinical phenotypes [8,9]. For instance, Leen et al. (2010) evaluated 57 patients with pathogenic variants in the *SLC2A1* gene and concluded that the type of mutation is related to the severity of intellectual disability in patients with GLUT1 deficiency syndrome. Mild intellectual disability was observed in 79 % of patients with a missense variant, while only 26 % of patients with nonsense, frameshift, splice, translation initiation or multiple exon deletion variants had mild intellectual disability. Motor disorders were observed less frequently in patients with missense variants (63 %) than in patients with nonsense, frameshift, splice or translation initiation variants (88 %) [10].

Yang et al. (2011) studied 53 patients diagnosed with GLUT1 deficiency syndrome and initially classified them according to a clinical scoring system for grading phenotypic severity, categorizing patients into minimal, mild, moderate and severe severity [11,12]. A comparison of the type of heterozygous pathogenic *SLC2A1* variants in the four groups revealed that missense variants were predominantly present in the mild and moderate clinical categories. Splice variants, nonsense, insertions and exon deletions occurred almost exclusively in the moderate and severe clinical categories [12].

The variant found in our patient and his phenotype do not corroborate the genotype/phenotype correlation previously described, since it had a frameshift variant and a mild atypical phenotype [8].

The main limitation of the study is the absence of functional studies related to the described variant and the poor literature about mild cases of GLUT1 deficiency. Later descriptions of this variant in other affected individuals may corroborate for a better genotype/phenotype correlation.

## 4 Conclusion

Our study enriches the *SLC2A1* gene mutation spectrum and emphasizes the importance of molecular genetic study in patients with neuropsychomotor developmental delay. Furthermore, we draw attention to a possible discrepancy between the genotype/phenotype correlation previously established in relation to patients with GLUT1 deficiency syndrome and carriers of the new variant described.

## Funding

Financiamento e Incentivo à Pesquisa (Fipe/HCPA).

## Ethics approval

All procedures followed were in accordance with the ethical standards of the responsible committee on human experimentation (institutional and national) and with the Helsinki Declaration of 1975, as revised in 2000.

This study was approved by the Ethics and Research Committee number 5327 of the Hospital de Clínicas de Porto Alegre (CAEE: 65118222.5.0000.5327).

## Patient consent statement

Informed consent was obtained from the legal guardian of the patient included in the study.

## CRediT authorship contribution statement

**Lívia Maria Ferreira Sobrinho:** Writing – review & editing, Writing – original draft, Visualization, Supervision, Software, Methodology, Investigation, Formal analysis, Data curation, Conceptualization. **Thiago Oliveira Silva:** Writing – review & editing, Conceptualization. **Lilia Farret Refosco:** Writing – review & editing, Visualization, Validation, Supervision, Methodology, Investigation, Conceptualization. **Soraia Poloni:** Writing – review & editing, Investigation, Conceptualization. **Fabiano Oliveira Poswar:** Writing – review & editing, Visualization, Validation, Supervision, Investigation, Formal analysis, Conceptualization. **Carolina Fischinger Moura de Souza:** Writing – review & editing, Visualization, Validation, Supervision, Investigation, Formal analysis, Data curation, Conceptualization. **Fernanda Sperb-Ludwig:** Writing – review & editing, Visualization, Validation, Supervision, Investigation, Formal analysis, Data curation, Conceptualization. **Ida Vanessa Doederlein Schwartz:** Writing – review & editing, Visualization, Validation, Supervision, Software, Resources, Project administration, Methodology, Investigation, Funding acquisition, Formal analysis, Data curation, Conceptualization.

## Declaration of competing interest

No conflict of interest to declare.

## Data Availability

Data will be made available on request.

## References

[1] Klepper J., Akman C., Armeno M. (2020). Glut1 deficiency syndrome (Glut1DS): state of the art in 2020 and recommendations of the international Glut1DS study group. Epilepsia Open..

[2] Diaz J., Fonseca A.G., Arboleda R. (2021). Case report: the Association of Wilson Disease in a patient with Ataxia and GLUT-1 deficiency. Front. Pediatr..

[3] Vivo De, Darryl C., Jacobson R.I. (1991). Defective glucose transport across the blood-brain barrier as a cause of persistent hypoglycorrhachia, seizures, and developmental delay. N. Engl. J. Med..

[4] Kramer J., Smith L. (2021). Ketogenic diet in Glut 1 deficiency through the life cycle: pregnancy to neonate to preschooler. Child Neurol. Open..

[5] Koch H., Weber Y.G. (2019). The glucose transporter type 1 (Glut1) syndromes. Epilepsy Behav..

[6] Richards S., Aziz N., Bale S. (2015). Standards and guidelines for the interpretation of sequence variants: a joint consensus recommendation of the American College of Medical Genetics and Genomics and the Association for Molecular Pathology. Genet. Med..

[7] Hildebrand M.S., Damiano J.A., Mullen S.A. (2014). Glucose metabolism transporters and epilepsy: only GLUT 1 has an established role. Epilepsia.

[8] Jacobson R.I. (2021). Discovery of Glut 1 deficiency syndrome: cerebrospinal fluid inspiration and serendipity. Pediatr. Neurol..

[9] Olivotto S., Duse A., Bova S.M. (2022). Glut1 deficiency syndrome throughout life: clinical phenotypes, intelligence, life achievements and quality of life in familial cases. Orphanet J. Rare Dis..

[10] Leen Wilhelmina G. (2010). Glucose transporter-1 deficiency syndrome: the expanding clinical and genetic spectrum of a treatable disorder. Brain.

[11] Kaufmann P., Shungu D.C., Sano M.C., Jhung S., Engelstad K., Mitsis E., Mao X., Shanske S., Hirano M., DiMauro S., De Vivo D.C. (2004). Cerebral lactic acidosis correlates with neurological impairment in MELAS. Neurology.

[12] Yang H., Wang D., Engelstad K., Bagay L., Wei Y., Rotstein M., Aggarwal V., Levy B., Ma L., Chung W.K., De Vivo D.C. (2011). Glut1 deficiency syndrome and erythrocyte glucose uptake assay. Ann. Neurol..

